# Dr. Warren Welch

**Published:** 1883-07

**Authors:** 


### OBITUARY.

DR. WARREN WELCH died Monday, May 28th, 1883, in Baltimore City where he had been engaged in the practice of dentistry for many years. Dr. Welch studied his profession with the late Dr. Cyrenius O. Cone, and graduated at the Baltimore College of Dental Surgery in the class of 1851-52

Of a retiring and somewhat sensitive disposition, but at the same time genial and friendly with those who knew him best, Dr. Welch never associated with his professional brethren in association meetings and intercourse to any great degree, and hence was not so generally known among the profession as many others. Notwithstanding, on account of his ability as a practitioner and his character as a gentleman, he merited and retained the respect of those older members of the dental profession who for many years were his contemporaries. As a dentist, Dr. Welch was a practitioner of ability and skill, and save for his retiring disposition, would have been one of the most successful in Baltimore City, where he practiced during the entire course of his professional life, with the exception of a short period spent in Europe. He was the originator of a "Nerve Paste" which for many years has had quite a local reputation, and also was an accomplished mechanical dentist, especially in block, continuous-gum and metal work. His judgment could be relied upon, and in private life he merited the esteem of all who knew him. "*Qualis vita finis ita.*"

